# Association of iron rim lesions with brain and cervical cord volume in relapsing multiple sclerosis

**DOI:** 10.1007/s00330-021-08233-w

**Published:** 2021-09-22

**Authors:** Claudia E. Weber, Julia Krämer, Matthias Wittayer, Johannes Gregori, Sigurd Randoll, Florian Weiler, Stefan Heldmann, Christina Roßmanith, Michael Platten, Achim Gass, Philipp Eisele

**Affiliations:** 1 Department of Neurology, Medical Faculty Mannheim and Mannheim Center for Translational Neurosciences (MCTN), University of Heidelberg, Theodor-Kutzer-Ufer 1 – 3, 68167 Mannheim, Germany; 2 Department of Neurology With Institute of Translational Neurology, University Hospital Münster, Albert-Schweitzer-Campus 1; Gebäude A1, Westturm, Ebene 5, 48149 Münster, Germany; 3 Mediri GmbH, Eppelheimer Straße 113, 69115 Heidelberg, Germany; 4 Fraunhofer MEVIS, Am Fallturm 1, 28359 Bremen, Germany

**Keywords:** Multiple sclerosis, Magnetic resonance imaging, Spinal cord

## Abstract

**Objectives:**

In multiple sclerosis (MS), iron rim lesions (IRLs) are indicators of chronic low-grade inflammation and ongoing tissue destruction. The aim of this study was to assess the relationship of IRLs with clinical measures and magnetic resonance imaging (MRI) markers, in particular brain and cervical cord volume.

**Methods:**

Clinical and MRI parameters from 102 relapsing MS patients (no relapses for at least 6 months, no contrast-enhancing lesions) were included; follow-up data obtained after 12 months was available in 49 patients. IRLs were identified on susceptibility-weighted images (SWIs). In addition to standard brain and spinal cord MRI parameters, normalised cross-sectional area (nCSA) of the upper cervical cord was calculated.

**Results:**

Thirty-eight patients had at least one IRL on SWI MRI. At baseline, patients with IRLs had higher EDSS scores, higher lesion loads (brain and spinal cord), and lower cortical grey matter volumes and a lower nCSA. At follow-up, brain atrophy rates were higher in patients with IRLs. IRLs correlated spatially with T1-hypointense lesions.

**Conclusions:**

Relapsing MS patients with IRLs showed more aggressive MRI disease characteristics in both the cross-sectional and longitudinal analyses.

**Key Points:**

*• Multiple sclerosis patients with iron rim lesions had higher EDSS scores, higher brain and spinal cord lesion loads, lower cortical grey matter volumes, and a lower normalised cross-sectional area of the upper cervical spinal cord.*

*• Iron rim lesions are a new lesion descriptor obtained from susceptibility-weighted MRI. Our data suggests that further exploration of this lesion characteristic in regard to a poorer prognosis in multiple sclerosis patients is warranted.*

## Introduction

In multiple sclerosis (MS), magnetic resonance imaging (MRI) allows the requested demonstration of dissemination in time and space [1] and plays an important role in disease monitoring. Even though focal T2-hyperintense lesions represent a characteristic hallmark of MS pathology, T2 lesion load does not correlate with disability [2].

Recently, there has been an increasing interest in “chronic active” MS lesions that are associated with higher disease severity and ongoing tissue destruction as an imaging biomarker of disease progression [3–8]. Histopathologically, chronic active lesions are characterised by a self-sustained low degree of chronic inflammation, active myelin breakdown, neurodegeneration, axonal loss, and progressive tissue matrix damage driven by a rim of iron-laden activated microglia/macrophages and reactive astrocytes at the lesion edge [4, 9]. Previous combined MRI/post-mortem studies demonstrated that these pathophysiological conditions can be visualised as hypointense rims on susceptibility-weighted imaging (SWI) [4, 6, 7], termed “iron rim lesions” (IRLs) [6, 10]. While previous studies reported an association between IRLs and brain volumes [6], the relationship between IRLs and spinal cord volumes has not been investigated until now.

As part of the central nervous system, the spinal cord represents an eloquent site of MS pathology and 80–90% patients show focal lesions and tissue abnormalities on spinal cord MRI [11]. Previous MRI studies demonstrated the association of spinal cord atrophy and disability [12–16]. Spinal cord atrophy is routinely measured as the cross-sectional area (CSA) at high cervical levels that provide reproducible results and are least affected by movements artefacts [11, 17].

The aims of this study were (i) to characterise differences of brain and spinal cord MRI readouts and (ii) to investigate the 1-year progression of brain and spinal cord atrophy in MS patients with IRLs compared to patients without those lesions. We hypothesise that IRLs correlate with clinical disability and MRI markers, in particular brain and cervical cord volume.

## Materials and methods

### Patients

#### Cross-sectional investigation

**Fig. 1:**
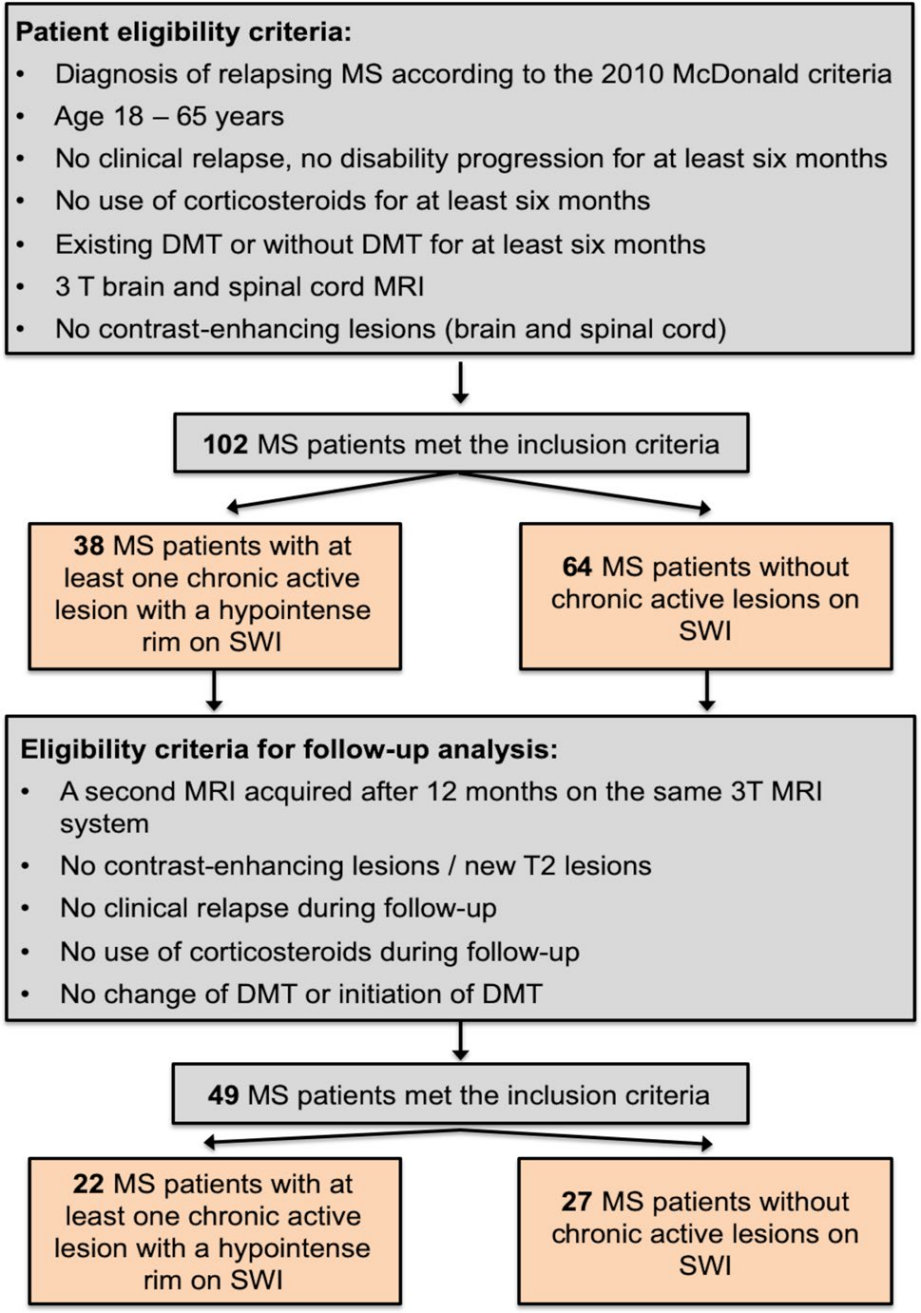
Flowchart summarising how patients were selected for the study. Abbreviations: DMT, disease-modifying therapy; MS, multiple sclerosis; SWI, susceptibility-weighted imaging

We retrospectively screened our database to identify patients diagnosed with relapsing MS according to the 2010 diagnostic criteria [1] fulfilling the following inclusion criteria (Fig. 1): (i) 18–65 years of age; (ii) a 3-T brain and spinal cord MRI dataset acquired in a single session including a 3D magnetization-prepared rapid acquisition gradient-echo (MPRAGE) sequence (brain) covering the upper portion of the cervical spinal cord (C1/2), a 3D fluid-attenuated inversion recovery (FLAIR) dataset (brain), SWI (brain), and sagittal T2-weighted cervical spinal cord images; (iii) no clinical relapse, no disability progression for at least 6 months; (iv) existing disease-modifying therapy (DMT) or without DMT for at least 6 months, (v) no use of corticosteroids 6 months prior; and (vi) no acute contrast-enhancing lesions (brain and spinal cord MRI). Exclusion criteria were presence of neurological conditions other than MS, cardiovascular or respiratory disease, history of brain/spinal cord trauma, severe cord compression, and missing or unsatisfactory MRI data. On the day of the MRI examinations, all patients underwent a comprehensive clinical assessment including the Expanded Disability Status Scale (EDSS) by trained neurologists.

#### Longitudinal investigation

For all patients included in the cross-sectional study, we screened if patients were eligible for a longitudinal follow-up substudy. To be included in the longitudinal study, patients had to fulfil the following inclusion criteria (Fig. 1): a second MRI acquired after 12 months follow-up on the same 3-T MRI system using identical imaging parameters, absence of acute contrast-enhancing or new T2-hyperintense lesions, no clinical relapse, and no use of corticosteroids and no change of DMT (in case patients were on DMT at baseline) or initiation of DMT (in case patients were without DMT) during the follow-up period. Confirmed disability accumulation was defined as disability increase from study baseline, measured by EDSS (increase of ≥ 1.0 points if baseline EDSS was ≤ 5.5 or an increase ≥ 0.5 for baseline EDSS > 5.5) [18]. Definition of converting to secondary progressive MS (SPMS) consisted a baseline EDSS ≥ 4.0 and an increase ≥ 1.0 points in patients with EDSS ≤ 5.5 or an increase ≥ 0.5 in patients with EDSS ≥ 6.0 [19].

### Magnetic resonance imaging

In all patients, MRI was performed on a 3-T MR system (MAGNETOM Skyra, Siemens Healthineers, 20-channel head coil) using a standardised protocol including a brain 3D MPRAGE sequence covering the upper portion of the cervical spinal cord (echo time (TE) = 2.49 ms, repetition time (TR) = 1900 ms, inversion time (TI) = 900 ms, field of view (FOV) = 240 mm, spatial resolution = 0.9 × 0.9 × 0.9 mm^3^), a brain 3D FLAIR dataset (TE = 398 ms, TR = 5000 ms, TI = 1800 ms, FOV = 240 mm, resolution = 0.5 × 0.5 × 0.9 mm^3^), brain SWI (TR = 27 ms, TE = 20 ms, FOV = 220 mm, slice thickness (ST) = 1.5 mm, voxel size 0.9 × 0.9 × 1.5 mm) acquired after injection of gadoterate meglumine at a standard dose of 0.1 mmol/kg, T1-weighted images (TR = 225 ms, TE = 2.5 ms, FOV = 220 mm, ST = 3 mm, voxel size 0.7 × 0.7 × 3.0 mm) acquired 10 min after contrast injection, and sagittal T2-weighted images (TE = 108 ms, TR = 3.500 ms, FOV = 300 mm, ST = 3.0 mm) with full coverage of the cervical cord.

### Spinal cord analysis

**Fig. 2:**
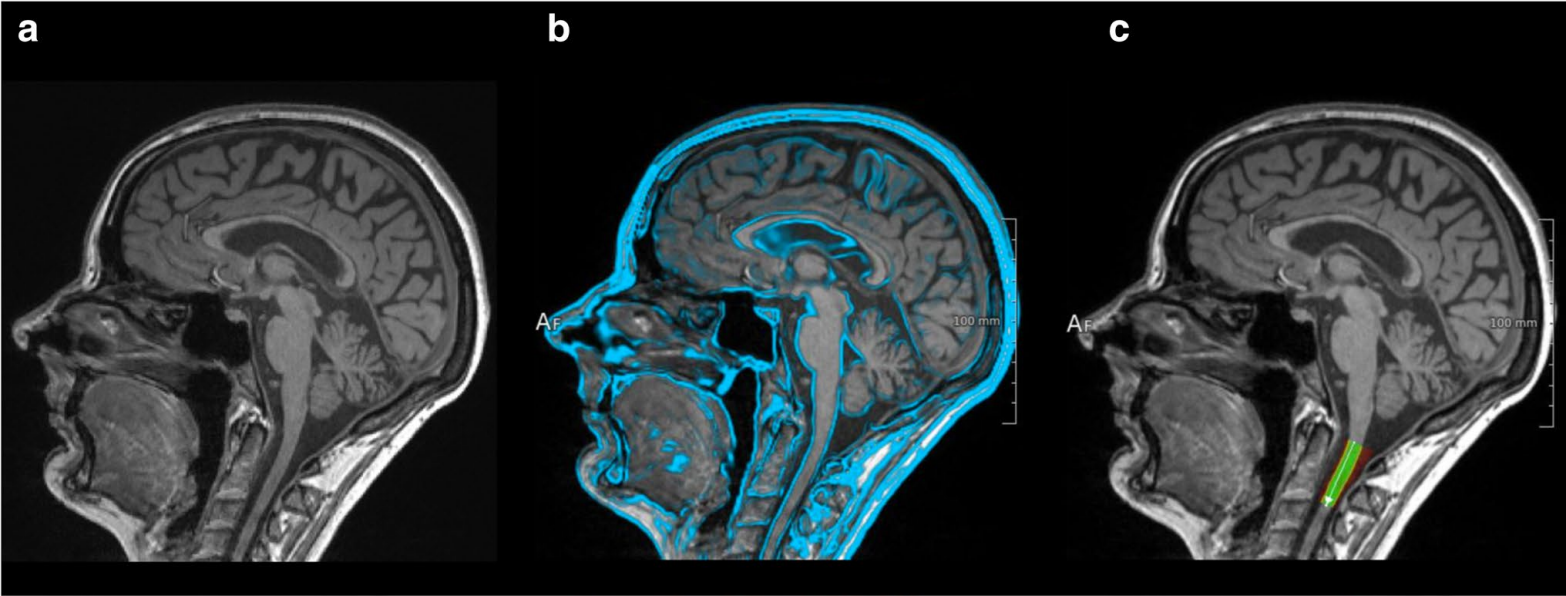
Calculation of the cross-sectional area (CSA) from a 3D magnetization-prepared rapid acquisition gradient-echo (MPRAGE) sequence (**a**) covering the upper portion of the cervical cord. (**b**) An atlas template image is registered to the subjects’ MPRAGE image. (**c**) Extraction of the mean area along the segmented spine

Brain MPRAGE datasets were uploaded to the “mTRIAL 3.3” cloud service (mediri GmbH, www.mediri.com) that includes pseudonymization, automatic identification, and image quality assessment. Mean CSA at the C1/2 level in all patients was determined using the integrated component MSAAutoSegmentationCLI 1.1.7 (Fraunhofer MEVIS). The software uses a fully automated atlas-based approach for detection and segmentation of the upper cervical spinal cord from 3D brain MPRAGE images. In short, an atlas template is registered non-linearly to the subjects’ image and spine segment definitions contained in the atlas template are transformed to the image. The pre-defined segments serve as a control for an iterative watershed-based segmentation, followed by fitting a bi-modal Gaussian mixture model with partial volume modelling to the histogram for volume measurement of the cord. The mean CSA is then obtained as the total cord volume divided by the cord length (Fig. 2) [20, 21]. Baseline (and if available follow-up) CSA was normalised (nCSA) as suggested previously [12].

Cervical cord lesions on sagittal T2-weighted images were identified by an experienced reader. Lesions were outlined with a semi-automated assistance using the drawing tool of MRIcroGL (https://www.mccauslandcenter.sc.edu/mricrogl/home). In cases no spinal cord lesions were detected, an empty lesion mask was generated as suggested previously [22]. A heat map was computed to demonstrate relative probabilities of spinal cord lesions. Therefore, sagittal T2-weighted images and lesion masks (baseline only) were registered to the PAM50 spinal cord template, co-registering all data to a common space [23] using the Spinal Cord Toolbox (version 4.0; http://spinalcordtoolbox.com) [24]. Qualities of the registrations were approved by visual inspection. The relative probabilities of the lesions occurring at a given voxel location were determined by combining the corresponding masks over all patients.

### Brain MRI analysis

Baseline brain tissue volumes, normalised for subject head size, were estimated with the automated model-based segmentation tool, SIENAX [25, 26], part of FSL (http://fsl.fmrib.ox.ac.uk/fsl/fslwiki/SIENA) [27], while percentage brain volume change (PBVC) during follow-up was calculated using the SIENA software [25, 26]. To correct for misclassification of T1-weighted grey matter volume in the presence of high T1-weighted hypointense lesion volume, T1-weighted hypointense lesions were filled with the mean intensity value of the normal-appearing white matter present in the same slice of the lesion. Calculation of deep grey matter (DGM) volumes was performed with the subcortical brain segmentation tool FSL FIRST. Since the main focus of our study was the spinal cord, only the average of the total DGM volume (sum of bilateral putamen, caudate, globus pallidus, thalamus) and thalamic volume was included in our statistical analysis [13].

**Fig. 3:**
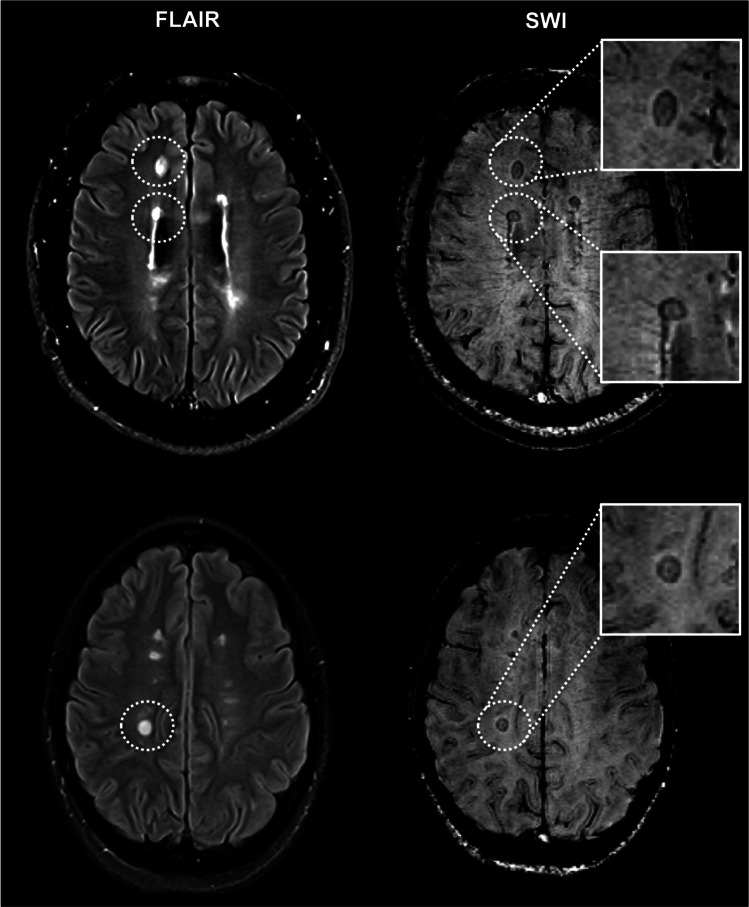
Representative examples of iron rim lesions (IRLs). Fluid-attenuated inversion recovery (FLAIR; left) and susceptibility-weighted images (SWI; right) demonstrate multiple IRLs (encircled and magnification)

SWI images were evaluated for the presence of IRLs by consensus by two MRI MS trial and diagnostic readers with each > 15 years of MR reading experience, unaware of the results of other MRI readouts, as described previously [28]: areas of hypointense ring-like signals at the lesion edge, encircling it fully or partially (see Fig. 3). Brain T2-hyperintense, T1-hypointense, and iron rim lesion volumes (LV) were quantified using the drawing tool of MRIcroGL. Heat maps representing relative probabilities of lesion subtypes (T2-hyperintense, T1-hypointense, IRLs) were computed over all patients. Therefore, baseline images were registered to the MNI152 template using the automated FSL FLIRT tool. The same registration was applied to the lesion masks. Computation of the heat maps was performed analogous to the spinal cord.

### Statistical analysis

Statistical analysis was performed with R (version 4.0.2). Group comparisons (MS patients with versus without IRLs) of dichotomous variables across the study groups were analysed using chi-square tests, while unpaired variables were assessed using the Mann–Whitney *U* test, since most of the variables were non-normally distributed. Level of significance was adjusted according to the Benjamini–Hochberg transformation despite the exploratory nature of the analysis.

For multivariate regression analysis of influence variables for the nCSA at the C1/C2 level, the variable was z-transformed to achieve normal distribution. We performed a linear model regression to model the influence of clinical and imaging variables on the z-transformed nCSA using group (rim-positive vs. rim-negative), volume of IRLs, number of IRLs, DMT, high efficiency DMT (fingolimod, natalizumab), disease duration, cervical LV, T1- and T2-LV, age, gender, and EDSS at baseline as potential covariates. We applied an automated modelbuilding algorithm using MASS–package of R using Akaike information criterion (AIC) as goodness of fit indicator. The Kolmogorov–Smirnov test was used to confirm a normal distribution of the residuals.

Finally, to elucidate the clinical relevance of the findings, we conducted a correlation analysis for the variables EDSS at baseline and nCSA C1/C2 at baseline with other baseline variables. As most of the continuous parameters were non-normally distributed, we used Spearman’s rank correlations. As many tests were conducted, we adjusted the significance level according to the Benjamini–Hochberg transformation despite the exploratory nature of the analysis.

We then performed repeated measures ANOVAs analysing the influence of group and time point and the interaction of it on the variables reassessed for follow-up. Imaging measures that were reassessed in follow-up were referenced to the baseline in order to calculate the difference to the baseline.

### Standard protocol approvals, registrations, and patient consents

The local ethics committee (2017-830R-MA) approved this study. Patient consent was waived due to the retrospective nature and lack of patient interaction.

## Results

### Cross-sectional investigation

Overall, 102 patients met the inclusion criteria. After lesion identification and classification, thirty-eight (37%) patients had at least one IRL on SWI (mean 1.8 lesions per patient; range 1–7), whereas in the remaining sixty-four patients, no IRLs were found on SWI. All IRLs showed matching hypointensity on T1-weighted MRI.

The groups did not differ in terms of gender (patients with IRLs: 74% female versus patients without IRLs: 75% female; *p* = 0.93), DMT (79% versus 80%; *p* = 1.0), high efficiency DMT (natalizumab, fingolimod; 37% versus 24%; *p* = 0.34), age (40.95 ± 10.12 versus 35.67 ± 10.65 years; *p* = 0.38), and disease duration (8.58 ± 8.85 versus 3.48 ± 3.29 years; *p* = 0.12), whereas patients with IRLs had a significant higher median EDSS score (3.0 versus 1.0; *p* < 0.001; Table 1).

**Table 1:** Mann–Whitney *U* tests for unpaired variables for group comparisons

	MS patients with IRLs	MS patients without IRLs	*W*	*p* value	Adjusted *p* value
Mean age, years (SD)	40.95 (10.12)	35.67 (10.65)	867.5	0.01	0.38
Mean disease duration, years (SD)	8.58 (8.85)	3.48 (3.29)	818.0	0.004	0.12
Median EDSS, baseline (range)	3 (0–6.5)	1 (0–6)	523.5	< 0.001	< 0.001
Mean nCSA C1/2, mm^2^ (SD)	65.14 (7.9)	70.66 (7.49)	1697.0	< 0.001	0.02
Mean volume, NAGM, mL (SD)	739.74 (56.81)	790.89 (53.57)	1792.0	< 0.001	0.002
Mean volume, NAWM, mL (SD)	722.43 (61.58)	745.16 (49.54)	1509.0	0.04	1
Mean volume, thalamus, mL (SD)	14.62 (2.20)	15.52 (1.67)	1578.5	0.01	0.29
Mean volume, DGM, mL (SD)	33.45 (4.52)	35.32 (3.55)	1542.0	0.02	0.58
Mean T2-LV, mL (SD)	9.39 (11.99)	2.87 (5.34)	480.5	< 0.001	< 0.001
Mean T1-LV, mL (SD)	2.29 (3.25)	0.35 (0.72)	482.5	< 0.001	< 0.001
Mean number of cervical lesions (SD)	2.34 (1.66)	0.98 (1.29)	641.0	< 0.001	< 0.001
Mean cervical LV, mL (SD)	0.96 (0.82)	0.26 (0.42)	510.0	< 0.001	< 0.001

Abbreviations: *DGM*, deep grey matter; *EDSS*, Expanded Disability Status Scale; *IRLs*, iron rim lesions; *LV*, lesion volume; *NAGM*, normal-appearing grey matter; *NAWM*, normal-appearing white matter; *nCSA*, normalised cross-sectional area; *SD*, standard deviation

**Fig. 4:**
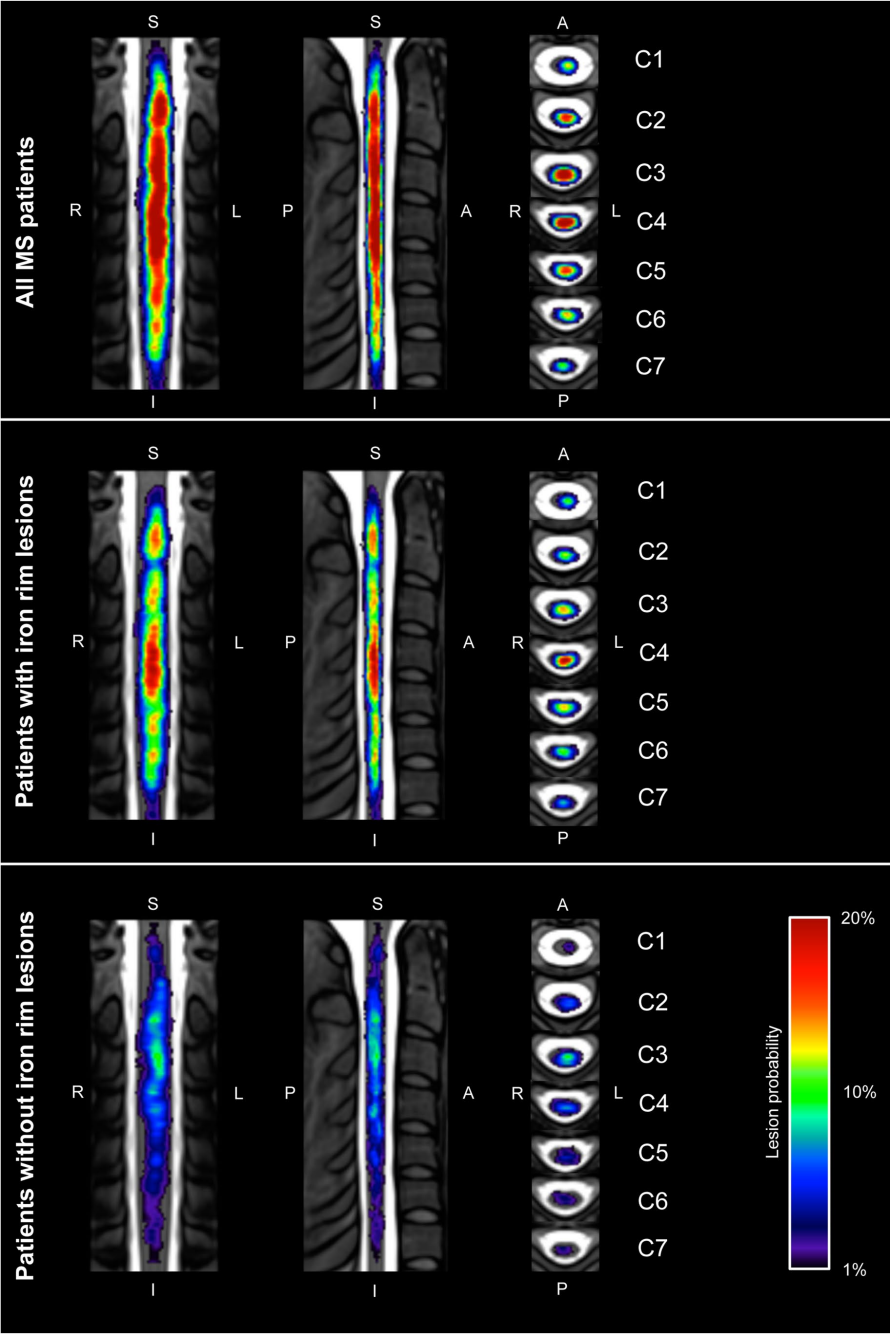
Heat map representation of relative probabilities of spinal cord lesions computed over all patients (top row), patients with iron rim lesions (middle row), and patients without iron rim lesions (bottom row) superimposed on the PAM50 spinal cord template. A, anterior; I, inferior; L, left; P, posterior; R, right; S, superior

**Fig. 5:**
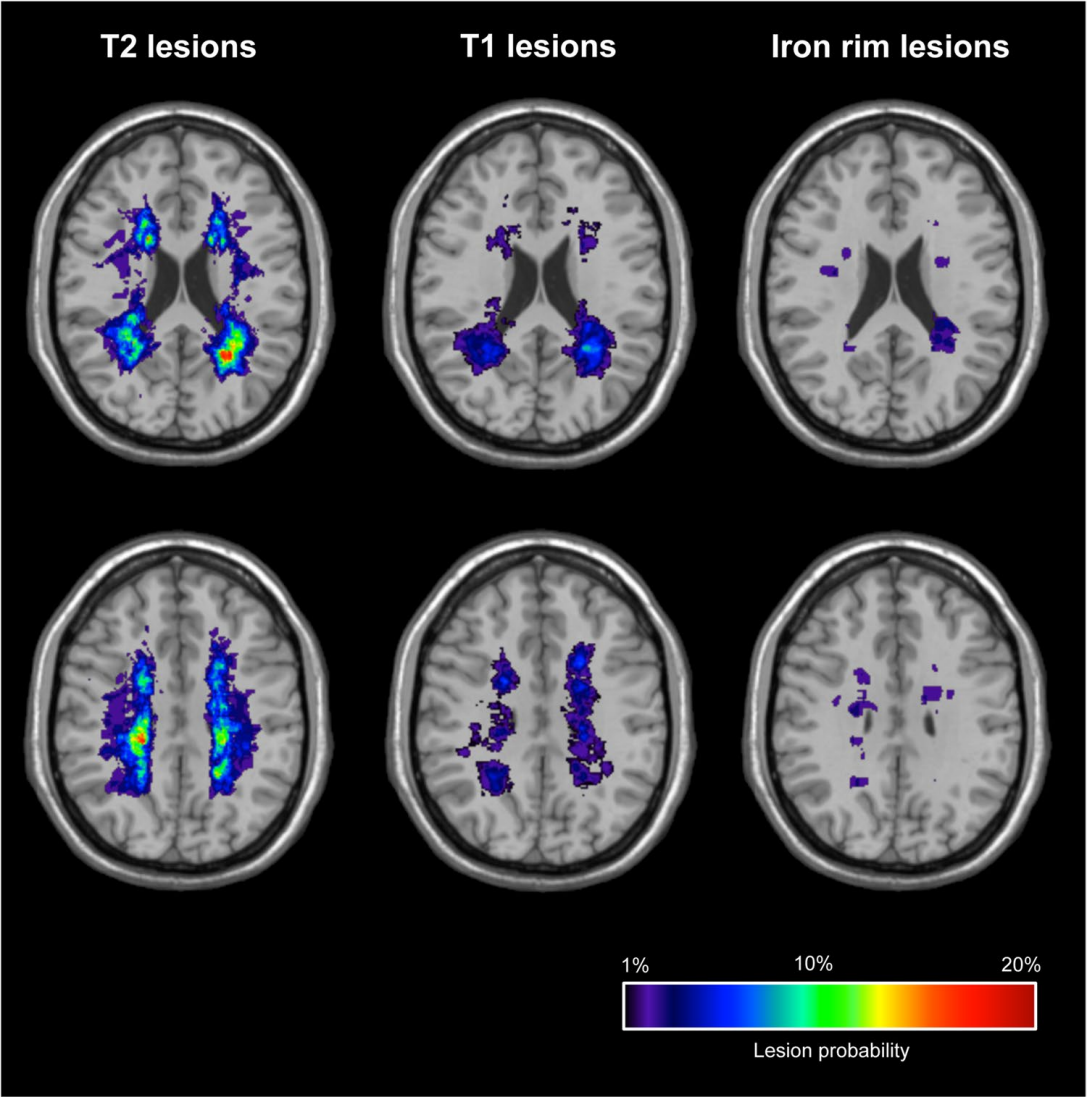
Heat map representation of relative probabilities of T2-hyperintense, T1-hypointense, and iron rim lesions computed over all patients included in the study superimposed on a MNI152 template

On baseline MRI, patients with IRLs had a significant lower mean nCSA C1/2, lower NAGM volumes, a higher T2- and T1-LV, a higher number of cervical lesions, and a higher cervical LV (Table 1). Figure 4 demonstrates a heat map of relative probabilities of spinal cord lesions. Relative probabilities of T2-hyperintense, T1-hypointense, and IRLs for all study participants are shown in Fig. 5.

### Linear model for the z-transformed CSA C1/C2

The automated model building using MASS package in R with AIC as goodness of fit criterion resulted in a linear model including the variables group, gender, disease duration, cervical LV, and DMT as independent variables and the z-transformed nCSA C1/C2 at baseline as the dependent variable. All other variables were removed from the model, as they did not improve the amount of explanation of the variance. The linear model was highly significant (*p* < 0.001) and showed a moderate explanation of variance (*R*^2^ = 0.2, adjusted *R*^2^ = 0.16). Independent variables of the model are shown in Table 2. The model showed no multicollinearity. Residuals were not correlated to the independent variables and normally distributed by visual inspection of the histogram though the Shapiro–Wilk test showed tendencies towards skewness. Results of the correlation analysis between baseline EDSS and nCSA C1/C2 with the other baseline variables are shown in Table 3.

**Table 2:** Linear model for the z-transformed normalised cross-sectional area at the C1/C2 level. **p < 0.01; *p < 0.5

	Estimate	*t* value	*p*	
Intercept	0.29	0.99	0.33	
Group	− 0.64	0.23	0.005	**
Gender	0.42	1.95	0.05	
Disease duration	− 0.04	− 2.47	0.02	*
Cervical lesion volume	0.24	1.56	0.12	
Disease-modifying therapy	− 0.35	− 1.5	0.14

**Table 3:** Multivariate correlation analysis; *p* values were adjusted using the Benjamini–Hochberg correction. Abbreviations: *adj.*, adjusted; *DGM*, deep grey matter; *EDSS*, Expanded Disability Status Scale; *IRLs*, iron rim lesions; *LV*, lesion volume; *nCSA*, normalised cross-sectional area; *NAGM*, normal-appearing grey matter; *NAWM*, normal-appearing white matter; *n.s.* not significant

	EDSS baseline		nCSA C1/2	
	*r*	adj. *p*	*r*	adj. *p*
Age	0.36	< 0.001	n.s	
Disease duration	0.43	< 0.001	− 0.24	0.002
EDSS baseline	-		− 0.31	0.003
nCSA C1/2	− 0.31	0.003	-	
Volume NAGM	− 0.26	0.01	0.2	0.05
Volume NAWM	0.39	< 0.001	0.34	< 0.001
Volume thalamus	− 0.26	0.01	0.41	< 0.001
Volume DGM	n.s		0.36	< 0.001
T2 LV	0.58	< 0.001	− 0.26	0.01
T1 LV	0.5	< 0.001	n.s	
IRL volumes	0.48	< 0.001	− 0.31	0.002
Cervical LV	0.56	< 0.001	− 0.21	0.04

### Longitudinal investigation

Forty-nine patients (48%) were included in the follow-up analysis. These included twenty-two patients with and twenty-seven patients without IRLs. Confirmed disability accumulation during follow-up was observed in seven patients (14%; 5/22 patients with IRLs; *p* = 0.3, repeated measures ANOVA for pre-post comparisons), and only one patient with IRLs fulfilled the definition of converting to SPMS [19]. During follow-up, PBVC was significantly higher in patients with IRLs (− 0.8 ± 0.6%) compared to patients without IRLs (− 0.34 ± 0.47%; *p* = 0.01). Differences of the cervical cord atrophy at the C1/2 level in patients with IRLs (− 2.44 ± 0.95%) and patients without IRLs (− 1.73 ± 0.96%) were not statistically significant (*p* = 0.6, repeated measures ANOVA for pre-post comparisons).

## Discussion

In this study, we investigated the association between IRLs with clinical measures and MRI readouts, in particular brain and cervical spinal cord volumes. There are several points noteworthy in this regard.

There has been an increasing interest in IRLs as a new imaging biomarker of disease progression indicating ongoing disease activity in the absence of contrast enhancement [3–6]. Even though the in-plane voxel dimensions of the axial SWI used in our study were larger compared to previous studies [6, 7], we found IRLs in ~ 40% of MS patients, a finding that is comparable to autopsy [29, 30] and MRI studies [28, 31], making SWI an interesting candidate for the detection of IRLs in a clinical setting. While post-mortem studies demonstrated that IRLs can be mainly found in progressive forms of MS [29, 32], our data and recent in vivo MRI studies show that a considerable amount of IRLs are also present in relapsing MS patients [3, 6]. Furthermore, our results are in line with previous studies that IRLs can mainly be found in patients with higher disease severity [6, 29]. In our study, IRL volumes and the EDSS correlated moderately supporting the view that IRLs could serve as a marker of disease severity [3–6]. Interestingly and in line with other studies [3, 5, 33], IRLs were present in patients despite DMT including fingolimod or natalizumab supporting the notion that IRLs evolve as a consequence of macrophage/microglia-mediated inflammation that may be independent of newly developing lesions [5, 6].

All IRLs showed matching hypointensity on T1-weighted MRI and appear to be a subgroup of hypointense T1 lesions, which have been previously described as markers of pronounced tissue matrix damage [34]. Several studies have shown the correlation between iron rims and slowly expanding lesions on T1-weighted MRI [6, 10]. In our cross-sectional analysis, patients with IRLs had higher EDSS scores, higher brain T2- and T1-LV, and lower volumes of the NAGM, a finding that is in line with recent studies [6]. Furthermore, cervical nCSA, a novel spinal cord volume marker, was also smaller in patients with IRLs who also showed higher cervical cord T2 LV. Based on the multivariate modelling approach, this was not merely an effect of the longer disease duration in patients with and without IRLs. Iron rim LV and number of IRLs did not show any contribution to the explanation of variance at all and were removed from the model. Therefore, we hypothesise that the sheer presence of IRLs and the underlying pathophysiology might have an effect on spinal cord atrophy independent of the number and volume of IRLs. Of note, we found a significant, yet moderate, correlation of the nCSA and EDSS scores that is comparable to previous studies [13, 16]. Interestingly, we also observed a significant correlation between cervical cord LV and disability, emphasising the relevance of demyelination in explaining disability [35] and underlining the heterogeneity and complexity of the disease. However, in our study, we used a sagittal T2-weighted sequence with a single long echo time for the detection of spinal cord lesion. Short tau inversion recovery sequences have a higher sensitivity [11]; therefore, the number of spinal cord lesions may be underestimated in our study.

In the 1-year longitudinal follow-up of 49 patients, the brain atrophy rate was higher in the IRL group. In line with previous observations, spinal cord atrophy rates were two to three times higher than brain atrophy [17]. Even though we found a trend of higher spinal cord atrophy rates in patients with IRLs, statistical significance was not reached, which is probably a result due to the relative short follow-up period. This is certainly an important limitation of our study. Another potential limitation is that SWIs were acquired after contrast injection. Therefore, interpretation of our results should be done cautiously.

The presence of IRLs appears to indicate MRI and clinical features that point to more aggressive MS characteristics. Both conventional lesion measures and indicators of irreversible tissue change (brain and cord atrophy) were more pronounced in patients with IRLs. SWI has been advocated to be used to increase diagnostic specificity in MS and new study data may emphasise this [36]. The results of this study suggest that SWI may have prognostic value. SWI sequences are available at 3-T and, with a lower sensitivity, at 1.5-T systems and therefore could be used in clinical imaging. Previous longitudinal studies reported a slow expansion of IRLs [4, 6, 10] and that iron rims persist and evolve over several years [4, 10]. Therefore, larger imaging intervals (e.g. 2–3 years) for monitoring IRLs may be sufficient for clinical purposes.

Future longitudinal studies also including primary and secondary progressive MS patients and longer observation periods would be highly interesting.

In conclusion, IRLs are associated with more aggressive MRI disease characteristics in relapsing MS.

## Abbreviations

DGM: Deep grey matter
DMT: Disease-modifying therapy
EDSS: Expanded Disability Status Scale
FLAIR: Fluid-attenuated inversion recovery
FOV: Field of view
IRLs: Iron rim lesions
LV: Lesion volume
MPRAGE: Magnetization-prepared rapid acquisition gradient-echo
MRI: Magnetic resonance imaging
MS: Multiple sclerosis
NAGM: Normal-appearing grey matter
NAWM: Normal-appearing white matter
nCSA: Normalised cross-sectional area
PBVC: Percentage brain volume change
ST: Slice thickness
SWI: Susceptibility-weighted imaging
TE: Echo time
TI: Inversion time
TR: Repetition time

## Declarations

### Guarantor

The scientific guarantor of this publication is Prof. Dr. Achim Gass, Department of Neurology, Medical Faculty Mannheim and Mannheim Center for Translational Neurosciences, University of Heidelberg, Theodor-Kutzer-Ufer 1 – 3, 68167 Mannheim, Germany.

### Statistics and biometry

One of the authors (Dr. Matthias Wittayer) has significant statistical expertise.

### Methodology

• retrospective

• iagnostic study

• performed at one institution
